# Metabolic impact of a nutrition education program for the promotion of fruit and vegetable consumption with people with severe mental disorders (DIETMENT)

**DOI:** 10.1186/s13104-022-06005-3

**Published:** 2022-03-29

**Authors:** Quintí Foguet-Boreu, Mireia Vilamala-Orra, Cristina Vaqué-Crusellas, Pere Roura-Poch, Montse Assens Tauste, Judit Bori Vila, Jose Manuel Santos-López, Ruben del Río Sáez

**Affiliations:** 1 Multidisciplinary Research Group in Mental Health, Department of Psychiatry, Vic University Hospital, 08500 Vic, Spain; 2 Department of Psychiatry, Vic University Hospital, 08500 Vic, Spain; 3 Faculty of Medicine, University of Vic – Central University of Catalonia, Vic, Barcelona Spain; 4 Research Group on Methodology, Methods, Models and Outcomes of Health and Social Sciences (M3O), Faculty of Health Sciences and Welfare, University of Vic – Central University of Catalonia, 08500 Vic, Spain; 5 Osonament – Osona Psychopedagogical Medical Center Foundation, 08500 Vic, Spain; 6 Department of Clinical Epidemiology, Vic University Hospital, 08500 Vic, Spain; 7 Mental Health and Social Innovation Research Group (SaMIS), University of Vic—Central University of Catalonia, 08500 Vic, Spain

**Keywords:** Metabolic, Nutrition education program, Severe mental disorders, Metabolic syndrome, Fruit and vegetables

## Abstract

**Objectives:**

The aim of this study is to determine the metabolic impact of a nutrition education program on metabolic parameters and the presence of metabolic syndrome (MetS).

**Results:**

Seventy-four patients were included (mean age, 48.7 years [Standard deviation, SD: 10.8], 55.4% men). The diagnoses of SMD were 37.8% schizophrenia and related disorders; 29.7% bipolar disorder; 25.7% depressive disorder; 4.1% personality disorders; and 2.7% obsessive compulsive disorders. Thirty-seven individuals were distributed in both the intervention group (IG) and the control group (CG). In the IG the presence of MetS was 56.3% and in the CG 46.7%, with no statistically significant difference (p = 0.309). At the end of the study, glomerular filtrate decreased in the IG, body mass index and abdominal perimeter increased in both groups, and there were no changes in metabolic parameters between the groups. Between the baseline and the end of the study, there was no increase in the number of patients diagnosed with MetS (14 at both points); and in the CG the increase was from 8 to 12 (p = 0.005). An intervention based on fruit and vegetable intake could prevent progression to MetS in individuals with SMD, decreasing the likelihood of cardiovascular disease.

*Trial registration* The trial was retrospectively registered on International Standard Randomised Controlled Trial Number (ISRCTN) Register on 11 March 2022 (ISRCTN12024347)

**Supplementary Information:**

The online version contains supplementary material available at 10.1186/s13104-022-06005-3.

## Introduction

Growing evidence suggests that diet combined with a healthy lifestyle has potential in the prevention and treatment of mental illness and may modify the effects of treatments [1]. The introduction of a diet rich in fruit and vegetables (F&V) is very beneficial for health, prevents cardiovascular diseases and some types of cancer, and in general is associated with a higher quality of life and good mental health [2–6]⁠.

The international recommendation of five portions of F&V a day has also been shown to be beneficial for mental health [6]. Despite this, evidence suggests that eating F&V may offer modest benefits in reducing cardiovascular risk factors [7].

In the case of people with severe mental disorder (SMD), there is a difficulty in maintaining healthy lifestyle habits, including the consumption of F&V. Several implemented intervention strategies have been aimed at people with SMD and focused on increasing the practice of physical activity and improving diet quality [8, 9]. Our group has recently published a study employing the Transtheoretical Model to promote healthy eating behaviors [10]. The study aimed to investigate changes in fruit and vegetable intake and the motivation to do so among people with SMD after participating in a food education program based on the stages of change model. The authors reported positive results in the short and long term, not only in fruit intake but also in an increase in the awareness and disposition of people with SMD towards health care. In this article, we analyze the metabolic impact of that intervention in terms of metabolic parameters and presence of metabolic syndrome (MetS).

## Main text

### Methods

A randomized community-based clinical trial was conducted between January 2019 and September 2020. The study protocol, methodological aspects of data source and study population and results of the main study have been published in detail elsewhere [10, 11].⁠ We recruited participants with SMDs who were part of a psychosocial rehabilitation center (Osona Psychopedagogical Medical Center) located in Vic, Barcelona, Spain. This centre serves 160,821 people (according to the 2019 census) and attends to approximately 325 patients in a community rehabilitation area annually.

Based on previous studies, we calculated the sample size using the GRANMO sample calculation tool (https://www.imim.es). The original sample required 52 people per group to allow a detectable difference between groups, with an expected 20% percentage change in the proportion of participants who achieve the intake of five daily servings of food and vegetables, considering a significance of 0.05, 80% power in a unilateral contrast. A drop-out rate of 10% has been anticipated (ARCSINUS approximation).

We included individuals over the age of 18 with a clinical diagnosis of SMD who participated actively in a community rehabilitation program and excluded users of the residential services, those diagnosed with substance use disorder, dementia, relapse of mental disorder, moderate to severe intellectual development disorder, and individuals with a diet that contraindicates the consumption of F&V.

Seventy-four users completed baseline data collection. A person unconnected to the study performed the allocation concealment using the Zenon algorithm (equiprobable randomization 1:1 through R Software), considering the variables of age, gender, functionality, and primary mental health diagnosis.

The *Dietment* intervention program lasted 4 months (April to July 2019). It consisted of a food education strategy aimed at promoting the consumption of F&V and comprised 15 weekly group sessions (of 5–10 people) lasting 90 min each session. All sessions were conducted by the same dietitian-nutritionist. For more information about the intervention, see Vilamala-Orra et al. [10]. In the control group, three voluntary group sessions were offered to the participants’ relatives as support agents to facilitate the change of habits (60 min).

Data on metabolic parameters and MetS were collected at baseline and after a mean of 5 months postintervention.

### Variables

We selected the following variables to evaluate the metabolic impact of *Dietment* program: Socio-demographic variables: age, sex, marital status, level of education, and basic activities of daily living (BADL) support; Clinical variables: main psychiatric diagnosis (International Classification of Diseases, ICD-10); Anthropometric variables: height (cm), weight (kg), abdominal perimeter (cm), body mass index (BMI) [weight (kg)/size (m^2^)] and blood pressure (mmHg); Laboratory tests: basal glycaemia (mg/dl), glycated haemoglobin (%), total cholesterol (mg/dl), LDL cholesterol (mg/dl), HDL cholesterol (mg/dl), Triglycerides (mg/dl), Creatinine (mg/dl), Glomerular filtrate (ml/min). An additional file shows the technical details and measurement instruments (see Additional file 1). In addition, we determined the presence or absence of MetS. To define MetS, we used the following criteria: abdominal perimeter measurement of the Spanish population (94.5 cm in men and 89.5 cm for women); blood pressure (BP) > 130/85 mm Hg, triglyceride (TG) > 150 mg/dl; glycaemia > 100 mg/dl; HDL-cholesterol (< 40 mg/dl for men and 50 mg/dl for women). Subjects that had a prescribed medication for hypertension, dyslipidemia or impaired glucose tolerance/diabetes were considered as having the respective risk factors. For diagnosis of MetS, at least three abnormal components were required [12]. Other variables: physical activity (Brief Physical Activity Assessment Tool, (BPAAT)) and food consumption (General diet quality index) (Additional file 1).

### Statistical analysis

A descriptive analysis of the data was carried out. Qualitative variables were reported as frequencies and percentages, and quantitative variables were reported as averages and standard deviations (SD), if they were under normal distribution; when quantitative variables were not under normal distribution, the median and the interquartile range (IQR) were used. The comparison for categorical variables was performed using Pearson’s chi-square test or Fisher’s test if appropriate. The comparison of means was carried out by means of the Student’s T test (against dichotomous variables) or by means of an Anova test (against polychotomous variables), and if both were under normal distribution. Its parametric equivalents were used when the distribution of quantitative variables was asymmetric. The level of statistical significance used for all hypothesis tests was 5%. The analysis was carried out with the SPSS programme for Windows, version 26 (IBM International Group B.V. Amsterdam, Hollande).

### Results

Seventy-four patients were included in the study. The average age was 48.7 years (standard deviation, SD: 10.8), and 55.4% were men. The diagnoses of SMD were 37.8% schizophrenia and related disorders; 29.7% bipolar disorder; 25.7% depressive disorder; 4.1% personality disorders; and 2.7% obsessive compulsive disorders.

There were no differences between the two groups at basal evaluation in all the variables analysed (Table 1). In the intervention group the presence of MetS was 56.3% and in the control group 46.7%, with no statistically significant difference (p = 0.309). There were also no differences in all the MetS determinants between groups (Additional file 2: Table S1).

**Table 1:** Sample characteristics by group (N = 74)

Variables	Global population (N = 74)	Intervention group (n = 37)	Control group (n = 37)	p-value
Age (years), mean (SD)	48.7 (10.8)	49.8 (11.4)	47.7 (10.3)	0.358
Sex (men), n (%)	41 (55.4)	21 (56.8)	20 (54.1)	0.815
Marital status, n (%)				
Single	46 (63.0)	19 (52.8)	27 (73.0)	0.374
Separated or divorced	18 (24.7)	13 (36.1)	5 (13.5)	
Married or paired	9 (12.3)	4 (11.1)	5 (13.5)	
Level of education, n (%)				
No studies	1 (1.4)	1 (2.7)	–	0.168
Compulsory education	32 (43.2)	12 (32.4)	20 (54.1)	
Baccalaureate or training cycles	31 (41.9)	19 (51.4)	12 (32.4)	
Higher education	10 (13.5)	5 (13.5)	5 (13.4)	
AVD support, n (%)				
No support	39 (52.7)	19 (51.4)	20 (54.1)	0.955
Family or non-professional	20 (27.0)	10 (27.0)	10 (27.0)	
Professional	15 (20.3)	8 (21,6)	7 (18.9)	
Main psychiatric diagnosis, n (%)				
Schizophrenia & related disorders	28 (37.8)	12 (32.4)	16 (43.2)	0.535
Bipolar disorder	22 (29.7)	12 (32.4)	10 (27.0)	
Depressive disorder	19 (25.7)	10 (27.0)	9 (24.3)	
Personality disorders	3 (4.1)	1 (2.7)	2 (5.4)	
Obsessive compulsive disorders	2 (2.7)	2 (5.4)	–	
General diet quality index, mean (SD)	7.6 (2.9)	7.9 (2.9)	7.3 (2.8)	0.439
Physical activity, n (%)				
Insufficient	43 (58.1)	20 (54.1)	23 (62.2)	0.319
Sufficient	31 (41.9)	17 (45.9)	14 (37.8)	
Anthropometric variables, mean (SD)				
Weight (kg)	84.2 (18.1)	87.1 (17.5)	81.3 (18.5)	0.145
BMI (kg/m^2^)	29.6 (6.3)	30.6 (5.6)	28.7 (6.8)	0.100
Abdominal perimeter (cm)	103.8 (14.5)	105.1 (14.6)	102.6 (14.6)	0.164
Blood pressure (mm Hg)				
Systolic blood pressure	118.7 (15.1)	117.3 (15.1)	120.1 (15.3)	0.406
Diastolic blood pressure	82.5 (9.9)	81.2 (10.2)	84.0 (9.5)	0.360
Laboratory tests, mean (SD)				
Basal glycaemia (mg/dl)	100.2 (36.5)	95.5 (28.9)	104.8 (42.4)	0.258
Glycated haemoglobin (%)	5.8 (0.9)	5.6 (0.9)	5.9 (0.9)	0.286
Total cholesterol (mg/dl)	199.9 (39.0)	203.2 (42.4)	196.7 (35.6)	0.405
LDL cholesterol (mg/dl)	118.3 (33.8)	123.0 (36.5)	113.5 (30.5)	0.189
HDL cholesterol (mg/dl)	51.5 (12.2)	50.6 (13.3)	52,3 (11.0)	0.354
Triglycerides (mg/dl)	162.2 (111.2)	160.6 (86.9)	163.8 (132.1)	0.424
Creatinine (mg/dl)	0.9 (0.2)	0.9 (0.2)	0.9 (0.1)	0.557
Glomerular filtrate (ml/min)	82.7 (10.9)	82.0 (12.8)	83.4 (8.9)	0.903

After the intervention, the intervention group increased weight (87.0 to 89.0, p = 0.006), whereas no differences were observed in the control group (81.3 to 81.4, p = 0.432). A significant increase of BMI and abdominal perimeter were observed in both groups (Table 2). No differences in blood pressure, basal glycaemia, glycated haemoglobin, lipids and creatinine were observed. A statistically significant reduction of glomerular filtrate was observed in the intervention group (81.9 to 80.4, p = 0.022 (Table 2).

**Table 2:** Comparison before and after intervention of clinical and laboratory variables by group (N = 74)

Variables	Intervention group (n = 37)			Control group (n = 37)		
	Pre	Post	p-value	Pre	Post	p-value
Weight (kg)	87.0 (17.5)	89.0 (17.6)	0.006	81.3 (18.5)	81.4 (19.2)	0.432
BMI (kg/m2)	30.5 (5.9)	31.3 (5.9)	0.000	28.7 (7.0)	28.9 (6.9)	0.000
Abdominal perimeter (cm)	104.9 (15.2)	105.1 (15.8)	0.000	102.4 (15.0)	102.6 (14.3)	0.000
Blood pressure (mm Hg)						
Systolic blood pressure	115.6 (14.9)	118.1 (14.3)	0.108	119.4 (15.7)	122.8 (15.0)	0.206
Diastolic blood pressure	80.7 (10.3)	82.7 (9.8)	0.177	83.2 (9.9)	82.2 (9.7)	0.492
Basal glycaemia (mg/dl)	95.5 (28.9)	107.5 (60.6)	0.111	104.8 (42.4)	106.0 (53.6)	0.777
Glycated haemoglobin (%)	5.6 (0.9)	5.8 (1.2)	0.103	5.9 (0.9)	8.3 (9.0)	0.239
Total cholesterol (mg/dl)	203.2 (42.4)	201.2 (45.1)	0.618	196.7 (35.6)	195.1 (35.0)	0.668
LDL cholesterol (mg/dl)	123.0 (36.5)	120.8 (36.3)	0.662	113.5 (30.5)	109.8 (35.0)	0.352
HDL cholesterol (mg/dl)	50.6 (13.3)	51.6 (19.4)	0.182	52.3 (10.9)	55.4 (15.8)	0.306
Triglycerides (mg/dl)	160.6 (86.6)	167.4 (129.8)	0.953	163.8 (132.1)	163.2 (112.2)	0.802
Creatinine (mg/dl)	0.9 (0.2)	0.9 (0.2)	0.673	0.9 (0.1)	0.9 (0.1)	0.464
Glomerular filtrate (ml/min)	81.9 (12.7)	80.4 (13.2)	0.022	83.4 (8.9)	82.4 (8.4)	0.511

Data are expressed as mean and standard deviation (SD), unless otherwise stated

No statistically significant differences were found in physical activity and general diet between the intervention and control group. Neither before and after the intervention intra-groups.

After the follow-up period, in the intervention group 14 patients were diagnosed with MetS at baseline and at the end of the study, and in the control group the number increased from 8 patients at baseline to 12 at the end of the study (p = 0.005) (Table 3).

### Discussion

A food education program based on the stages of change model for severe mental disorders showed a reduction in the increase of incidence of MetS in the intervention group versus the control group. No significant differences were observed in metabolic parameters individually.

As far as we are aware, there is no study that has evaluated the effect of a nutrition education program for the promotion of fruit and vegetable consumption on the occurrence of MetS. Jones (2019) conducted a systematic review that showed that nutritional interventions led to significant weight loss [13]. In our study, weight, BMI, and abdominal perimeter worsened after the intervention in the intervention group. The latest published reviews assessing different metabolic aspects have mixed results. A recent Cochrane review evaluating interventions to prevent type 2 diabetes in patients with SMD in low- and middle-income countries was inconclusive [14]. A meta-analysis by Naslund et al. of lifestyle weight loss interventions for overweight and obesity in SMD patients, concluded that such interventions were effective, especially those lasting longer than 12 months, which have a clinically significant ≥ 5% weight loss at follow-up [15]. Moreover, another systematic review evaluating pharmacological and non-pharmacological treatments to improve glycaemic control in patients with SMD showed that behavioural interventions that have longer duration and included physical activity had greater effects on glycaemic than those without these characteristics [16]. On the other hand, we do not know the reasons for the decrease in glomerular filtration rate in the intervention group. Water supplementation in healthy patients helps to suppress the decline in kidney function over time but does not appear to decrease it [17].

Two studies with a wellbeing program with particular emphasis on healthy lifestyle promotion showed positive results. The first one, with a focus on psychoeducation, dietary advice and physical exercise, found a significant decrease in BMI (especially in patients with diabetes) and with a reduction of MetS prevalence after the educational program intervention [18]. The second one showed an improvement in levels of physical activity, smoking, diet and self-esteem with no changes in BMI [19]. The benefits of interventions that only incorporate physical exercise have no impact on weight, mental symptoms or quality of life [20], while those that combine exercise and diet, with or without other components, such as psychoeducation, reduced weight significantly [21], albeit with an effect that diminished over time.

In patients with SMD, there is a high prevalence of three factors that are key to the development of a MetS: sedentary lifestyles, unbalanced dietary patterns and medication-induced weight gain [22, 23]. As we have seen in our study, MetS without intervention increases with follow-up, similar to another study where MetS increased in a group of patients followed for 8 years [24]. Therefore, dietary modification can help to reduce the components of MetS, especially a reduction of fats (saturated, trans and cholesterol), sodium and sugars, which would help to prevent or control dyslipidemia, hyperglycemia and hypertension [25]. Although as Castro-Barquero et al. point out, a healthy diet should be based on a sum of dietary changes rather than a restriction of any single nutrient [26]. Previous studies have reported that SMD may lead to difficulties in daily activities, such as taking medication (86%) and preparing meals [27], which makes it even more difficult to follow healthy lifestyles Table 3.

**Table 3:** Metabolic syndrome determinants pre- and post-intervention by group

Variables	Intervention group			Control group		
	Pre	Post	p-value	Pre	Post	p-value
Waist circumference (cm)	104.9 (15.2)	105.1 (15.8)	0.766	102.4 (14.9)	102.6 (14.3)	0.771
Blood pressure (mm Hg)						
Systolic	115.6 (14.9)	118.1 (14.3)	0.108	119.4 (15.7)	122.8 (15.0)	0.206
Diastolic	80.7 (10.3)	82.7 (9.8)	0.177	83.2 (9.9)	82.2 (9.7)	0.492
Triglyceride (mg/dl)	164.6 (90.9)	165.6 (131.9)	0.953	159.5 (141.8)	163.1 (112.2)	0.802
Glycaemia (mg/dl)	92.0 (17.8)	108.3 (61.7)	0.111	104.8 (43.8)	106.0 (53.6)	0.777
HDL-cholesterol (mg/dl)	49.1 (14.3)	52.4 (19.3)	0.182	53.5 (10.8)	55.4 (15.8)	0.306
Metabolic syndrome (≥ 3), n (%)	14 (63.6)	14 (63.6)	0.072	8 (36.4)	12 (45.5)	0.005

Data are expressed as mean and standard deviation (SD), unless otherwise stated

The effectiveness of lifestyle interventions is clear when diet, exercise and psychoeducation are incorporated [28, 29]. Consequently, future interventions will have to take a multicomponent and multidisciplinary approach: to encompass different components (pharmacological and non-pharmacological) and to introduce the perspective of different health professionals (doctors, nurses, nutritionists, pharmacologists, psychologists, and sports specialists, among others). The cognitive, physical and mental conditions of people with SMD will also need to be taken into account in order to achieve a reduction of MetS.

## Conclusions

The presence of MetS increases the likelihood of cardiovascular disease and mortality [30]. While our results should be treated with caution given the small sample included in the study, they may indicate that an intervention based on fruit and vegetable intake prevents progression to a MetS in the short term.

## Limitations

A major strength of the study is its design, which allowed for comparison with a control group and a follow-up of participants at 12 months post-intervention. Another strong point was the inclusion of the nutrition education program for promoting healthy eating habits in a community rehabilitation service as an integral part of the individual’s recovery process. One of its weaknesses is the small sample size, which may limit the study’s power in detecting differences between groups. However, it is true that recruitment in intervention programmes of SMD patients is always difficult, and the drop-out rate from psychosocial treatment is around 13% [31].

## Supplementary Information

**Additional file 1: **Measurements and instruments

**Additional file 2: Table S1**. Basal distribution of Metabolic syndrome determinants by group (N = 62*)

## Data Availability

The datasets used and/or analyzed during the current study are available from the corresponding author on reasonable request.

## Abbreviations

DIETMENT: Program for the promotion of fruit and vegetable consumption with people with severe mental disorders
SMD: Severe Mental Disorders
IG: Intervention group
MetS: Metabolic Syndrome
CG: Control Group
BADL: Basic activities of daily living
ICD-10: International Classification of Disease
