# Supplementary material for: Metabolic impact of a nutrition education program for the promotion of fruit and vegetable consumption with people with severe mental disorders (DIETMENT)
Source: BMC Res Notes. 2022 Mar 29;15:122. doi: 10.1186/s13104-022-06005-3 (PMC8962513; doi:10.1186/s13104-022-06005-3)
Supplement: Supplementary file 1 — Additional file 1: Measurements and instruments [file 13104_2022_6005_MOESM1_ESM.pdf]

Additional file 1: Measurements

A mental health nurse performed the determination of blood pressure and anthropometric parameters. Blood pressure was determined with a semi-automatic and validated blood pressure monitor (OMRON M7 Intelli IT®. HEM-7361T-EBK), first obtaining the blood pressure in both arms and then choosing the highest one, and then taking another measurement in that arm to determine the arithmetic mean of the two measurements. Height and weight were measured with a portable measuring rod and digital scale (SECA 799® gmbh & co). The abdominal perimeter was measured locating the upper hip bone and the top of the right iliac crest, placing a measuring tape in a horizontal plane around the abdomen at the level of the iliac crest with a tape measure.

The laboratory parameters basal glycaemia was analyzed with the UV enzymatic (hexokinase method) technique (Beckman Coulter-AU5800®) and glycated haemoglobin (%) with the high-performance liquid chromatography (HPLC) technique (Hemoglobin analyzer HA8180 Menarini Diagnostics®). Total cholesterol, LDL cholesterol, HDL cholesterol, and triglycerides were determined by a colorimetric enzymatic technique (Beckman Coulter-AU5800®) obtained with mg/dl. Creatinine was determined by a kinetic colorimetric test (Jaffré method) (Beckman Coulter-AU5800®), with mg/dl. The glomerular filtrate was obtained with the CKD-EPI Creatinine Equation (Chronic Kidney Disease Epidemiology Collaboration), with ml/min).

Physical activity was determined by the Brief Physical Activity Assessment Tool (BPAAT) questionnaire validated for the Catalan population (1). It consists of two questions that allow the frequency and duration of physical activity to be measured at vigorous and moderate intensity for a week. The score system allows you to classify the person as “Sufficiently active" or "Insufficiently active".

The general diet quality index was based on the health food considerations contained in the consensus document drawn up by the Catalan Public Health Agency (ASPAC, 2019). The index groups the information collected in the consumer frequency questionnaire and the 24-hour dietary register. It consists of 19 items concerning diet fractionation, the quality of daily ingests and the frequency of consumption of major food groups. Each item admits a score of zero or one depending on whether the described premise is met. The total score can range from 0 to 19 points. A higher score represents higher diet quality (Annex 1). The designed index shows a correlation with the Mediterranean Diet Adherence Screener (MEDAS) of 0.644. MEDAS is considered the benchmark for assessing adherence to the Mediterranean diet pattern (2)., developed in the PREDIMED (Prevención con Dieta Mediterránea, Prevention with Mediterranean diet) study (3). The low-adherence results in the MEDAS test obtain an average score of 7.02 (SD: 2.6) in the diet quality index, and the good-adherence results an average score of 10.7 (SD: 2.6) ($p<0.001$). The area under the ROC curve is 0.832, indicating good discriminatory capability. In other words, the diet's general quality index ranks participants by 83.2% according to their adherence to the Mediterranean diet pattern based on the MEDAS test.

1. Puig Ribera A, Peña Chimenis O, Romaguera Bosch M, Duran Bellido E, Heras Tebar A, Solà Gonfaus M, et al. How to identify physical inactivity in primary care: validation of the Catalan and Spanish versions of 2 short questionnaires. Aten Primaria. 2012;44(8):485-93.
2. Schröder H, Fito M, Estruch R, Martinez-Gonzalez MA, Corella D, Salas-Salvado J, et al. A short screener is valid for assessing Mediterranean diet adherence among older spanish men and women. J Nutr. 2011;141(6):1140–1145.
3. Estruch R, Martínez-González MA, Corella D, Salas-Salvadó J, RuizGutiérrez V, Covas MI, et al. Effects of a Mediterranean-style diet on cardiovascular risk factors: a randomized trial. Ann Intern Med. 2006;145:1–11.

ANNEX 1. Diet quality index

Question	Score	Instrument
1. Meal frequency<br>Include between 5 and 6 ingests/day or intake every 3-4h<br>*Not include: single coffee, infusion or refreshment	+1p	R-24h
2. Nutritional quality of breakfast<br>It contains farinaceous*, natural lactic and/or fresh fruit	+1p	R-24h
3. Nutritional quality of lunch<br>It contains farinaceous*, vegetables, protein, fresh fruit or natural lactic	+1p	R-24h
4. Nutritional quality of dinner<br>It contains farinaceous*, vegetables, protein, fresh fruit or natural dairy	+1p	R-24h
5. Includes 1 ration of vegetables/day	+1p	R-24h
6. Includes a second vegetable ration	+1p	R-24h
7. Includes 2 pieces of fresh fruit per day	+1p	R-24h
8. Includes a third piece of fresh fruit	+1p	R-24h
9. Includes 1 to 3 rations of natural milk products per day	+ 1p	R-24h
10. Includes maximum 2 rations of meat, fish, egg and legumes per day. Alternating different food group.	+ 1p	R-24h
11. Include whole-grain farinaceous*	+1p	R-24h
12. Include legumes at least 3 rations per week	+1p	CFQ
13. Includes seafood at least 3 rations per week	+1p	CFQ
14. If you give priority to chicken meat, turkey or rabbit	+1p	CFQ
15. Includes eggs at least 3 rations per week	+1p	CFQ
16. Includes nuts at least 3 rations per week	+1p	CFQ
17. Water as the main drink	+1p	R-24h
18. Doesn't include sugar beverages.<br>Less than 1 ration per day.	+1p	R-24h
19. If it includes cookies, lactic desserts, pastries, sweet cereals.<br>Less than 2 rations.	+1p	CFQ

R-24h: 24-hour dietary record; CFQ: Consumer Frequency Quiz.
*We considered farinaceous: bread, pasta, rice, couscous, and legumes, potatoes and other tubers.
