# Supplementary material for: Metabolic impact of a nutrition education program for the promotion of fruit and vegetable consumption with people with severe mental disorders (DIETMENT)
Source: BMC Res Notes. 2022 Mar 29;15:122. doi: 10.1186/s13104-022-06005-3 (PMC8962513; doi:10.1186/s13104-022-06005-3)
Supplement: Supplementary file 2 — Additional file 2: Table S1. Basal distribution of Metabolic syndrome determinants by group (N = 62*) [file 13104_2022_6005_MOESM2_ESM.pdf]

Additional file 2: Table S1

Table S1. Basal distribution of Metabolic syndrome determinants by group (N=62*)

**Variables**	**Total (n=62)**	**Intervention group (n=32)**	**Control group (n=30)**	**P-value**
Waist circumference (WC)	84.7	88.9	80.6	0.257
Blood pressure (>130/85 mm Hg)	43.5	40.6	46.7	0.412
Triglyceride (>150 mg)	38.4	44.4	32.4	0.208
Glucose (>100 mg/dl)	23.3	22.2	24.3	0.526
HDL-cholesterol (less than 40 mg/dl for men and 50 mg/dl for women)	27.4	27.8	27.0	0.575
Pharmacological treatment for hypertension	25.7	24.3	27.0	0.500
Pharmacological treatment for dyslipidemia	10.8	16.2	5.4	0.131
Pharmacological treatment for impaired glucose tolerance/diabetes	12.2	10.8	13.5	0.500
Metabolic syndrome (≥3)	51.6	56.3	46.7	0.309

Note: results expressed in percentage of people in whom the determinant altered. *Twelve people did not have the values needed to calculate metabolic syndrome factors recorded and are therefore missing from this table.
